# *In Vivo* Attenuation of Antibody-Mediated Acute Renal Allograft Rejection by *Ex Vivo* TGF-β-Induced CD4^+^Foxp3^+^ Regulatory T Cells

**DOI:** 10.3389/fimmu.2017.01334

**Published:** 2017-10-16

**Authors:** Tao Liao, Youqiu Xue, Daqiang Zhao, Siwen Li, Mingyu Liu, Jingrong Chen, David Douglass Brand, Haofeng Zheng, Yannan Zhang, Song Guo Zheng, Qiquan Sun

**Affiliations:** ^1^Division of Kidney Transplantation, Department of Surgery, The Third Affiliated Hospital of Sun Yat-sen University, Guangzhou, China; ^2^Department of Clinical Immunology, The Third Affiliated Hospital of Sun Yat-sen University, Guangzhou, China; ^3^Division of Rheumatology, Milton S. Hershey Medical Center at Penn State University, Hershey, PA, United States; ^4^Key Laboratory of Gene Engineering of the Ministry of Education, State Key Laboratory of Biocontrol, School of Life Science of Sun Yat-sen University, Guangzhou, China; ^5^Research Service, Memphis VA Medical Center, Memphis, TN, United States

**Keywords:** antibody-mediated rejection, renal transplantation, regulatory T cell, TGF-β, macrophages

## Abstract

Antibody-mediated rejection (AMR) has emerged as the major cause of renal allograft dysfunction, and more effective strategies need to be explored for improving transplant outcomes. Regulatory T cells (Tregs), consisting of at least natural and induced Treg subsets, suppress effector responses at multiple levels and play a key role in transplantation tolerance. In this study, we investigated the effect of induced Tregs (iTregs) on preventing antibody-mediated renal injury and rejection in a mouse model. We observed that infusion of iTregs markedly attenuated histological graft injury and rejection and significantly improved renal allograft survival. iTregs exhibited a comprehensive ability to regulate immunological disorders in AMR. First, iTreg treatment decreased the levels of circulating antidonor antibody and the antibody deposition within allografts. Second, iTregs significantly reduced cell infiltration including CD4^+^ T cells (including Th1, Th17, and Tfh), CD8^+^IFN-γ^+^ cells, natural killer cells, B cells, and plasma cells, which are involved in the process of AMR. Our results also highlight a predominance of M1 macrophage infiltration in grafts with acute AMR, and M1 macrophage could be reduced by iTreg treatment. Collectively, our data demonstrate, for the first time, that TGF-β-induced Tregs can attenuate antibody-mediated acute renal allograft injury through targeting multiple effectors. Thus, use of iTregs in prevention of AMR in clinical practice could be expected.

## Introduction

The occurrence of current immunosuppression has markedly decreased T cell-mediated rejection of renal allografts; however, the incidence of antibody-mediated rejection (AMR) remains high and it is considered as the leading cause of renal allograft loss (1–3). 30–50% of acute rejection episodes and more than 60% of late graft dysfunction in renal transplants are due to the production of antidonor antibodies (2–4). Current strategies for controlling AMR are chiefly to remove antibodies and/or block complement activation including the use of plasmapheresis, intravenous immunoglobulin, rituximab, and bortezomib (5–8); however, these approaches appear to be only partially or transiently effective, or may result in severe complications (9). The difficulty in treating AMR has underscored the need to identify the mechanisms underlying antibody-mediated renal graft rejection and to develop more effective strategies to attenuate or prevent graft injury in AMR.

Regulatory T cells (Tregs), characterized by expression of CD4, CD25, and the transcription factor Foxp3, are well recognized with immunoregulatory function (10, 11). They consist of thymus-derived naturally occurring Tregs (nTregs) and induced Tregs (iTregs) that are induced *ex vivo* under specific condition with cytokines and antigen exposure (12). Tregs suppress effector responses through multiple mechanisms that include directly inhibiting CD4^+^ and CD8^+^ T cell activation and proliferation, suppressing B cell responses and antibody production as well as modulating macrophage and natural killer cell (NK cell) functions (13–16). Moreover, Tregs have been shown to play a critical role in transplantation tolerance (17). In most MHC-mismatched strain combinations of mice, the majority of kidney allografts are not rejected although moderate T cell and macrophage infiltration can be found in the grafts within 3 months after transplantation. A major tolerogenic mechanism was considered to be contributed by Tregs in the renal grafts since the depletion of these Foxp3^+^ Tregs resulted in mixed AMR (18), providing solid evidence that it was the Tregs that have prevented the development of AMR.

Although most studies have investigated the functional characteristics of nTregs in transplantation models, the weaknesses of nTreg cells in the clinical setting are obvious. nTregs originate in the thymus and their frequency is low. This makes it difficult to obtain sufficient numbers for clinical therapy. Although the expansion of nTregs *ex vivo* may overcome this problem, previous studies have demonstrated that nTregs are unstable after repeated expansions, particularly under inflammatory conditions (19–22). Due to the finding that iTregs can be induced *ex vivo* from naïve CD4^+^ cells in the presence of TGF-β (23, 24), obtaining sufficient numbers for Treg therapy is quite feasible. Not only do iTregs share many similar phenotypic and functional features with nTregs, but iTregs exhibit superior functional advantages when they are found in the presence of inflammatory conditions (22, 25–27). In addition, iTregs can be induced with specific donor antigens to become antigen-specific Treg cells that provide the additional advantages (28, 29).

This study was designed to determine if TGF-β-induced regulatory T cells (iTreg) could control antidonor antibody-mediated acute renal allograft rejection. To do this, we developed a mouse model of acute AMR and demonstrated that iTreg infusion markedly attenuates antibody-mediated renal rejection while also improving renal allograft survival, thus providing a novel prevention option for renal allograft recipients at high risk for AMR.

## Materials and Methods

### Ethics Statement

This study was carried out in accordance with the recommendations of the animal use protocol, which was approved by the Institutional Animal Care and Use Committee of Sun Yat-Sen University (Approve Number: 160520).

### Mice

Male adult C3H (H-2^k^) and BALB/c (H-2^d^) mice (Charles River, Beijing, China) weighing 25–30 g were donors and recipients, respectively. All animal experiments were performed in accordance with the Guide for the Care and Use of Laboratory Animals (National Institutes of Health publication no. 80-23, revised 1996).

### Generation of CD4^+^ iTreg

Splenocytes from BALB/c mice were used as a source for naïve CD4^+^CD62L^+^CD25^−^CD44^low^ T cells using a naïve CD4^+^ T cell isolation kit (Miltenyi Biotec, Auburn, CA, USA). Cells were cultured in RPMI 1640 medium supplemented with 10% heat-inactivated fetal bovine serum (Hyclone Laboratories), 100 U/ml penicillin, 100 mg/ml streptomycin, and 10 mM HEPES (Invitrogen). To induce Treg, naïve CD4^+^ T cells were incubated with anti-CD3/CD28 microbeads (one bead per five cells; Invitrogen) in the presence of IL-2 (50 U/ml; R&D Systems) with TGF-β (2 ng/ml; R&D Systems) for 3 days. Cells were harvested and then assessed for expression of Treg-associated markers (Figure S1 in Supplementary Material).

### Mouse Models

Full-thickness skin grafts (1 cm × 1 cm) from C3H donors were transplanted onto the backs of BALB/c recipient mice 4 days before kidney transplantation. Orthotopic kidney transplantation was performed. Briefly, kidney grafts were harvested from C3H mice and transplanted into the abdomens of presensitized BALB/c recipients by anastomosing the aorta, renal vein, and ureter of the graft to the recipient’s aorta, inferior vena cava, and bladder, respectively. Treatment of presensitized recipients with iTreg cells consisted of 3 × 10^6^ iTreg cells iv on day −5, −4, 0, and 3 after transplantation. We used this protocol since we and others previously reported that infusion of 1–3 × 10^6^ Treg cells in each mouse has resulted in an ideal effect on the prevention and treatment of arteriosclerosis, skin transplantation, acute graft-versus-host disease, arthritis, and lupus-like syndrome. Multiple injection could assure the sufficient Treg cell numbers. Day 0 was defined as the day of kidney transplantation.

### Circulating Donor-Specific Antibodies (DSA)

Circulating donor-specific IgG and IgM antibodies were assessed in recipient sera by flow cytometry. In short, recipient sera were incubated with C3H donor splenocytes at 37°C for 30 min, and washed cells were then incubated with FITC-labeled anti-mouse IgG (Abcam, Cambridge, England) and rhodamine red-conjugated anti-mouse IgM (Jackson ImmunoResearch Laboratories, West Grove, PA, USA) at 4°C for 1 h. Cells were analyzed by flow cytometry with results expressed as mean fluorescence intensity to reflect individual serum antidonor antibody levels.

### Histological Examination of Renal Tissue

On day 5 after kidney transplantation, the kidney grafts were harvested, formalin fixed and paraffin embedded. Staining with hematoxylin and eosin (H&E), periodic acid-Schiff, anti-C3d (R&D Systems, AF2655, 1:50) and anti-IgG antibody (Bethyl Laboratories, A90-116B, 1:500). The sections were examined for severity of rejection by light microscopy. Features of inflammation examined included peritubular capillary inflammatory cell infiltration, tubular injury as measured by the presence of acute epithelial damage as well as C3d and IgG deposition were measured according to the semiquantitative Banff scoring criteria: 0, absent; 1, mild; 2, moderate; and 3, prominent. Each stain was evaluated on four complete cross-sections. For immunofluorescence analyses, 6-µm tissue sections were cut from samples embedded in O.C.T. (SkuraFinetek). After drying, sections were fixed in 100% acetone for 10 min at 4°C, and then blocked with 10% BSA at room temperature for 30 min, following by staining primary antibodies at 4°C overnight. Alexa Fluor-conjugated secondary antibodies (Life Technologies) were used to detect specific murine antigens. The antibodies were as follows: mouse monoclonal antibody against mouse CD68 (abcam, ab955, 1:200), rat monoclonal antibody against mouse MHC class II (abcam, ab25333, 1:150), rabbit polyclonal antibody against mouse CD206 (abcam, ab64693, 1:100).

### TdT-Mediated dUTP Nick-End Labeling (TUNEL) Staining

Paraffin-fixed tissues were prepared by de-paraffin and rehydration and then subjected to TUNEL staining using the one step TUNEL apoptosis assay kit (Beyotime Biotechnology). The sections were visualized with DAPI.

### Flow Cytometry

Fresh kidney grafts were milled gently in phosphate-buffered saline (PBS) supplemented with 1% heat-inactivated fetal bovine serum using a needle on a 10 ml syringe before pressing through a 200-mesh nylon screen. Thereafter, the collected cells were stained with fluorochrome-conjugated Abs for CD45, CD3, CD4, CD8a, B220, CD138, CD11b, F4/80, CD86, CD206, CD279, CD278, CD185, CD49b, IFN-γ, IL-4, IL-17A, and analyzed by FACSCalibur flow cytometer using Cell Quest Software (BD Biosciences). For intracellular staining, such as Foxp3, IFN-γ, IL-4, and IL-17A, cells were stimulated with PMA and ionomycin for 5 h and brefeldin A for 4 h, then stained for surface markers and further fixed and permeabilized for intracellular or intranuclear staining. Data were analyzed with FlowJo Software (Tree Star, Ashland, OR, USA).

Antibodies used for flow cytometric analysis were purchased from BioLegend (San Diego, CA, USA): FITC-CD4 (RM4-5), PerCP/Cy5.5-IFN-γ (XMG1.2), APC-F4/80 (BM8), BV421-B220 (RA3-6B2), BV570-CD45 (30-F11), PE-IL-4 (11B11), APC-IL-17A (TC11-18H10.1), Alexa Fluor 647-Foxp3 (MF-14), PE-CD278 (15F9), PerCP/Cy5.5-CD279 (RMP1-30), APC-CD185 (L138D7), PE-CD138 (281-2), FITC-CD86 (GL-1), PE-CD206 (C068C2); eBioscience (San Diego, CA, USA): eFluor 450-CD8a (53-6.7), Alexa Fluor 700-CD3 (17A2), PE-CD49b; and BD Biosciences: PE-Cy7-CD11b (M1/70).

### Quantitative Reverse Transcriptase Polymerase Chain Reaction

Total RNA was extracted from flash-frozen kidney samples using TRIzol^®^ Reagent (Invitrogen) and reversely transcribed into cDNA using the Transcriptor First Strand cDNA Synthesis Kit (Roche LifeScience). The expression levels of iNOS, GM-CSF, and the housekeeping gene β-actin were quantified using the SYBR Green Reagents Kit (Roche LifeScience). The level of iNOS and GM-CSF was normalized to that of β-actin to calculate the 2^−ΔCt^ value. Data are presented as the mean ± SD from at least three independent experiments. Primers used are listed as follows: β-actin, 5′-AGGGAAATCGTGCGTGAC-3′ (forward) and 5′-CAAGAAGGAAGGCTGGAAA-3′ (reverse). iNOS, 5′-CCGCCGCTCTAATACTTA-3′ (forward), and 5′-TTCATCAAGGAATTATACAGGAA-3′ (reverse). GM-CSF: 5′-CAGTTGGAAGGCAGTATA-3′ (forward) and 5′-AAATAAATATAATGGTCCC TATCA-3′ (reverse).

### Statistical Analysis

The Mann–Whitney *U* test was utilized to compare the differences between two groups. Graft survival among groups was compared using the *log-rank test*. Differences with *p*-values < 0.05 were considered to be statistically significant.

## Results

### A Model of Acute AMR of Renal Grafts in Presensitized Recipients

**Figure 1:**
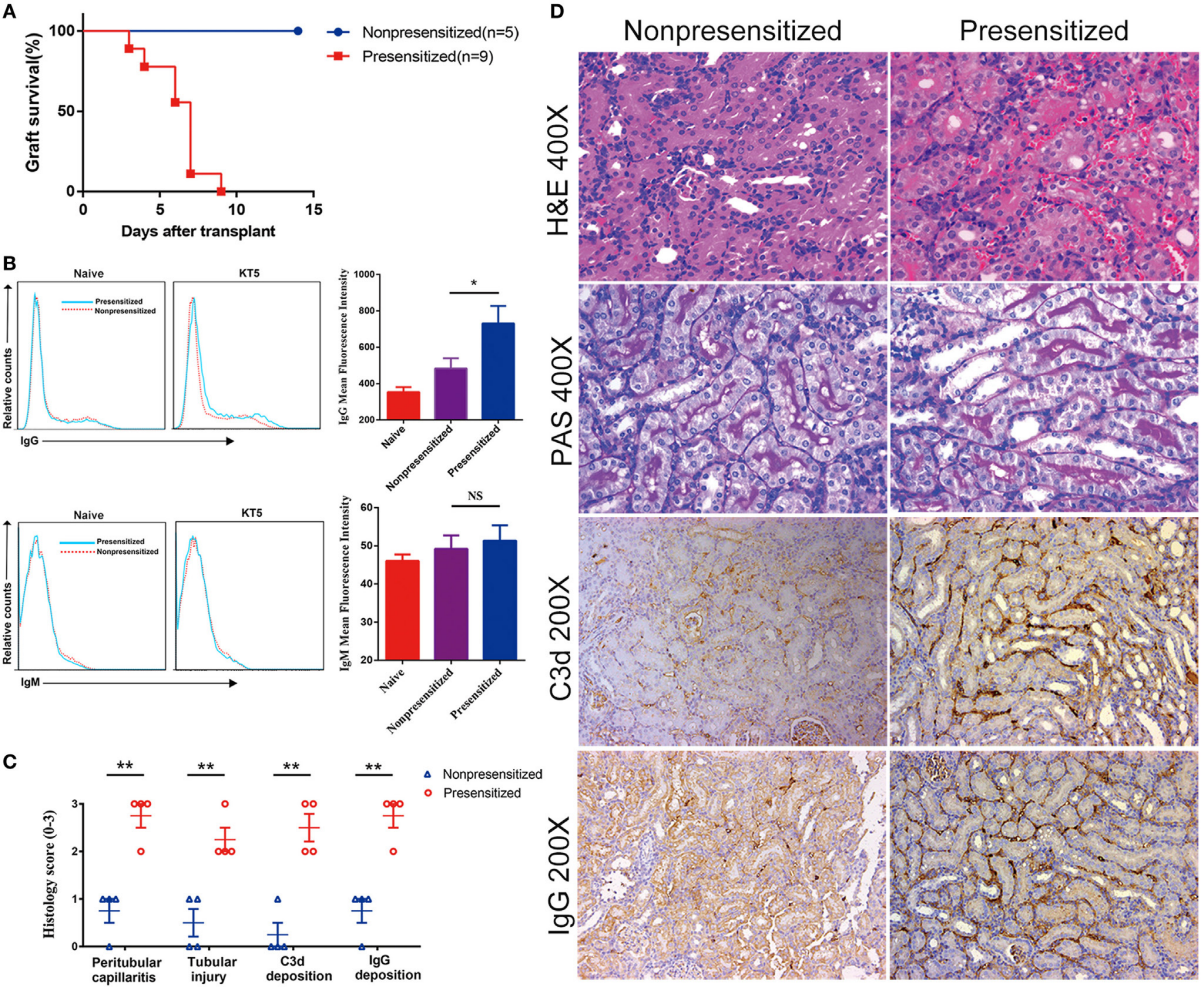
Skin presensitization before kidney transplantation induces acute antibody-mediated rejection. Renal allografts were obtained at 5 days post operation. **(A)** Survival of allografts from donors; **(B)** levels of antidonor-specific antibodies (IgG and IgM) before operation and at 5 days post kidney transplantation (KT5) by immunofluorescence and flow cytometry. Date are expressed as mean fluorescence intensity; **(C)** histological changes evaluated by the semiquantitative Banff scoring criteria: 0, absent; 1, mild; 2, moderate; and 3, prominent; **(D)** histologic evaluation of renal allografts posttransplant. Hematoxylin and eosin staining showing interstitial vasculitis, hemorrhage, and edema. Periodic acid-Schiff (PAS) staining showing tubular necrosis. Anti-C3d antibody and anti-IgG antibody staining showing the deposition of C3d and IgG (*p < 0.05, **p < 0.01).

Several different laboratories have previously established a mouse model of acute antibody-mediated renal allograft rejection using skin grafts from presensitized mice (30, 31). Using this model, we transplanted C3H kidney allografts into BALB/c recipient mice ± presensitization 4 days after they were grafted with skin from C3H donors. Graft survival, levels of antidonor IgG and IgM antibodies, and histology were examined at the indicated time points. Allografts in presensitized recipients were rejected rapidly (6.2 ± 1.8 days), whereas those not presensitized which received grafts survived indefinitely (Figure 1A). Naïve kidneys were removed at the time of transplantation, rendering recipient host survival dependent on allograft function. Among presensitized recipients, at the time of rejection (5 days posttransplant), levels of anti-DSA of the IgG class rather than IgM were found to be significantly higher compared with non-presensitized recipients (Figure 1B). Furthermore, histological features of grafts suggest that they are consistent with acute AMR, including evidence of (1) interstitial vasculitis, hemorrhage and edema; (2) tubular necrosis; and (3) intragraft deposition of C3d and IgG (Figure 1D). These features are significantly more severe than those seen in the non-presensitized group (Figure 1C).

### iTreg Treatment Prevents AMR and Injury while Also Improves Renal Allograft Survival in Presensitized Recipients

**Figure 2:**
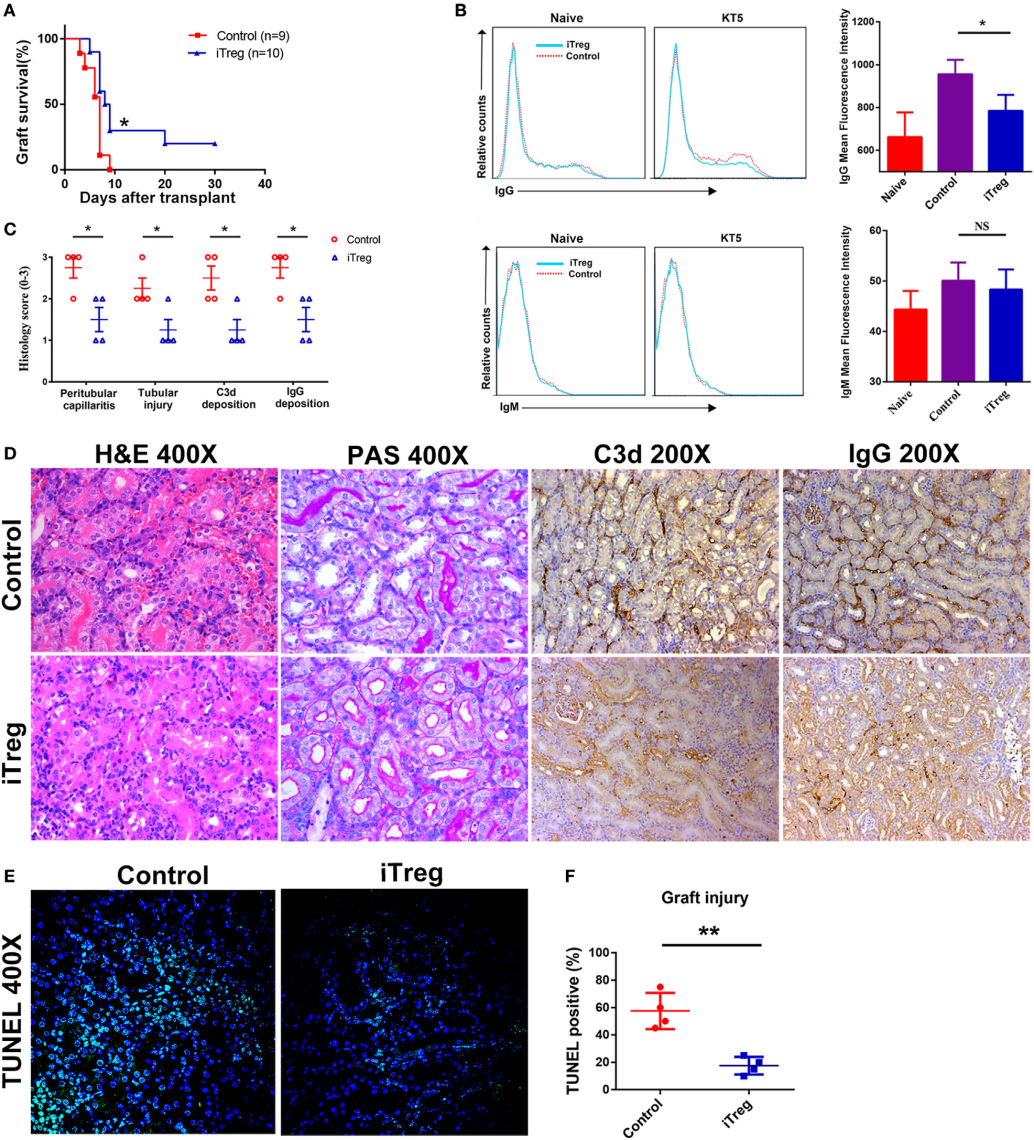
iTreg treatment attenuates renal allograft rejection and injury and prolongs allograft survival. Renal allografts were obtained at 5 days post operation. **(A)** Survival time; **(B)** levels of antidonor-specific antibodies (IgG and IgM) before operation and at 5 days post kidney transplantation (KT5) by immunofluorescence and flow cytometry. Data are expressed as mean fluorescence intensity; **(C)** histological changes evaluated by the semiquantitative Banff scoring criteria: 0, absent; 1, mild; 2, moderate; and 3, prominent; **(D)** histologic evaluation of a rejected renal allograft posttransplant. The graft sections were stained with hematoxylin and eosin, periodic acid-Schiff (PAS), anti-C3d antibody, and anti-IgG antibody; **(E)** cell apoptosis posttransplant using TdT-mediated dUTP nick-end labeling (TUNEL) assay; **(F)** statistical analysis of graft injury (*p < 0.05, **p < 0.01).

We have utilized the severe antibody-mediated allograft rejection model with presensitized renal recipients as described above to assess whether the immunomodulatory function of iTreg might prevent the rejection. We found that presensitized recipients given iTreg therapy have significantly improved kidney allograft survival. About 30 and 20% of grafts from iTreg-treated mice survived on 14 and 30 days, while all grafts were rejected before day 9 in the PBS-treated group (Figure 2A). iTreg-treated recipients exhibited lower levels of DSA-IgG compared with controls, although levels of DSA-IgM were not changed significantly (Figure 2B). Moreover, iTreg treatment also significantly reduced inflammatory cell infiltration in peritubular capillaries and tubular necrosis of grafts, as well as intragraft deposition of C3d and IgG as measured by histological examination (Figures 2C,D). Furthermore, TUNEL assay revealed that iTreg treatment markedly alleviated graft injury in presensitized recipients (Figures 2E,F).

### iTreg Treatment Reduces CD4^+^ Cells (including Th1, Th17, and Tfh) and CD8^+^IFN-γ^+^ Cells Infiltration in the Grafts

**Figure 3:**
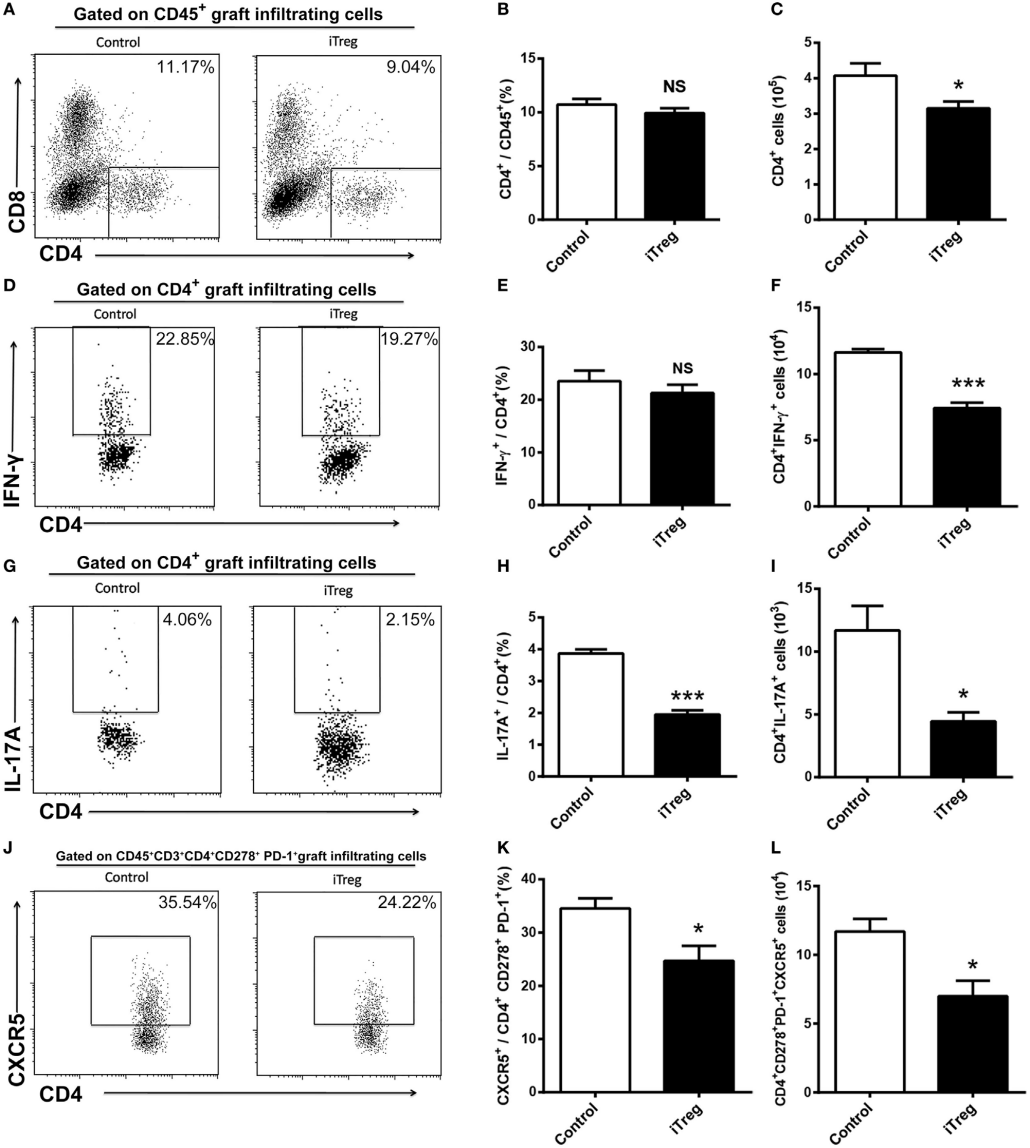
iTreg treatment reduces CD4^+^ cells including Th1, Th17, and Tfh infiltration in renal grafts. Renal allografts were obtained at 5 days post operation. **(A)** CD4^+^ cells among CD45^+^ cells; **(B)** relative percentages of CD4^+^ cells among the CD45^+^ cells; **(C)** absolute number of CD4^+^ cells; **(D,E)** IFN-γ^+^ cells among the CD4^+^ cells; **(F)** CD4^+^IFN-γ^+^ cells; **(G,H)** IL-17A^+^ cells among the CD4^+^ cells; **(I)** CD4^+^IL-17A^+^ cells; **(J,K)** CXCR5^+^ cells among the CD4^+^CD278^+^PD-1^+^ cells; **(L)** CD4^+^CD278^+^PD-1^+^CXCR5^+^ cells. Data are shown as mean ± SD of four transplants (*p < 0.05, ***p < 0.001).

We used flow cytometry to examine the numbers, frequencies, and phenotypes of CD4^+^ T cells in allografts obtained 5 days after transplantation. We determined that Th1 (CD4^+^IFN-γ^+^), Th2 (CD4^+^IL-4^+^), Th17 (CD4^+^IL-17A^+^), and Tfh (CD4^+^CD278^+^PD-1^+^CXCR5^+^) cells were present in the grafts. As shown in Figure 3, iTreg treatment significantly reduced the absolute numbers of CD4^+^ cells although it had no effect on relative percentages of CD4^+^ cells among the CD45^+^ cells in the grafts (Figures 3A–C). Among CD4^+^ T cell subsets, iTreg treatment also decreased the absolute numbers of Th1, Th17, and Tfh cells, as well as lowering Th17 and Tfh cell percentages, though it had no change on the relative percentage of IFN-γ^+^ cells among CD4^+^ cells (Figures 3D–L). Cells of the Th2 phenotype were undetectable by flow cytometry in this model.

**Figure 4:**
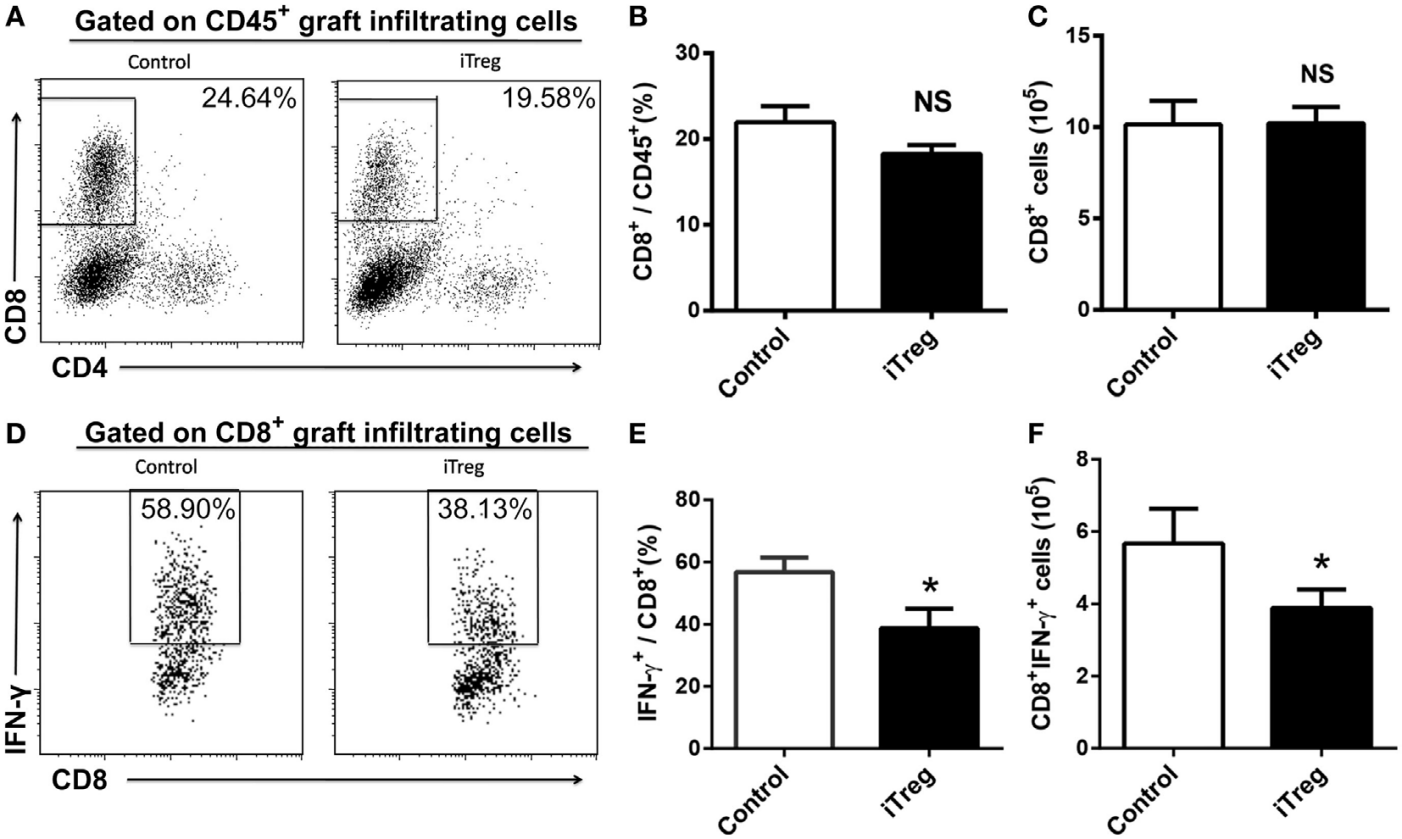
iTreg treatment reduces CD8^+^IFN-γ^+^ cells infiltration in renal grafts. Renal allografts were obtained at 5 days post operation. **(A)** CD8^+^ cells among CD45^+^ cells; **(B)** relative percentages of CD8^+^ cells among the CD45^+^ cells; **(C)** absolute number of CD8^+^ cells; **(D)** IFN-γ^+^ cells among the total CD8^+^ population; **(E)** IFN-γ^+^ cells among the CD8^+^ cells; **(F)** CD8^+^IFN-γ^+^ cells. Data are shown as mean ± SD of four transplants (*p < 0.05).

In addition to CD4^+^ subsets, we also evaluated the infiltration of total CD8^+^ and Tc1 (CD8^+^IFN-γ^+^) subsets in grafts from each group. iTreg treatment had neither effect on absolute numbers of CD8^+^ cells nor on its percentages among CD45^+^ cells in the grafts (Figures 4A–C). Nonetheless, iTreg treatment reduced both absolute number of Tc1 and its percentage among total CD8^+^ cells (Figures 4D–F). It is likely that iTreg treatment only affects Tc1 activation and cytokine production.

### iTreg Treatment Reduces M1 Macrophage and NK Cell Infiltration in the Grafts

**Figure 5:**
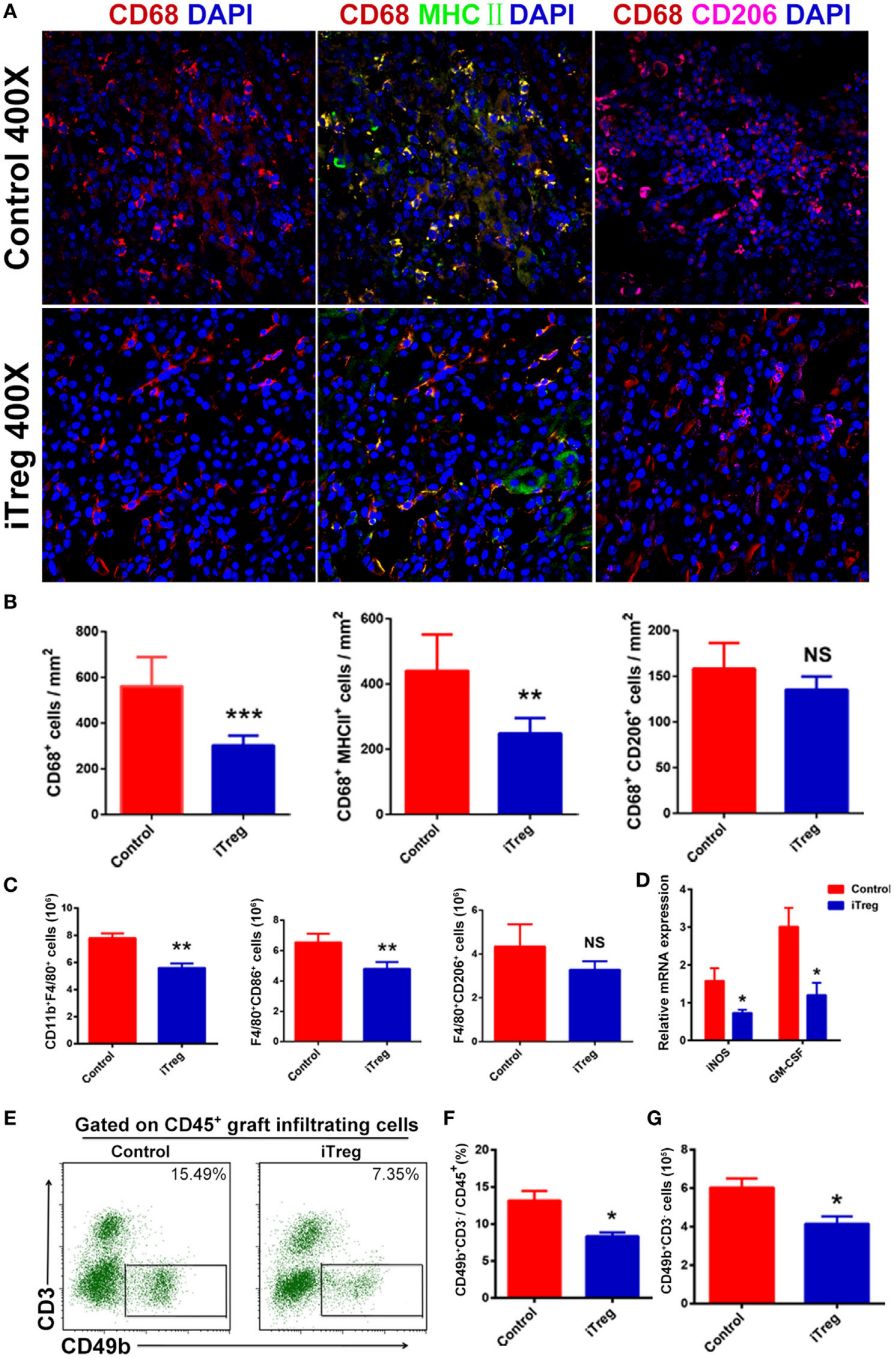
iTreg treatment reduces M1 macrophage and natural killer cell (NK cell) infiltration in renal allografts after transplantation. Renal allografts were obtained at 5 days post operation. **(A)** Immunofluorescence staining of graft-infiltrating macrophages, macrophages were determined by staining for DAPI and CD68. Merged fluorescence (yellow) of double staining for CD68 (red) and MHCII (green) determined M1. Merged fluorescence (amaranth) of double staining for CD68 (red) and CD206 (purple) determined M2; **(B)** cell counts from immunofluorescence staining in showing CD68^+^ (M), CD68^+^MHCII^+^ (M1), and CD68^+^CD206^+^ (M2) cells per square millimeter. Data are shown as mean ± SD of three independence samples; **(C)** absolute number of macrophages (F4/80^+^CD11b^+^) including M1 (F4/80^+^CD86^+^) and M2 (F4/80^+^CD206^+^) in grafts by flow cytometry. Data are shown as mean ± SD of four grafts; **(D)** the mRNAs expressions of iNOS and GM-CSF detected by qPCR; **(E)** detection of NK cells (CD49b^+^CD3^−^) among CD45^+^ cells in grafts by flow cytometry. **(F)** Relative percentage of CD49b^+^CD3^−^ cells among the CD45^+^ cells in the grafts. Data are shown as mean ± SD of four transplants. **(G)** Absolute number of CD49b^+^CD3^−^ cells infiltration in the grafts. Data are shown as mean ± SD of four grafts (*p < 0.05).

Macrophages consist of two subsets including classically activated macrophage (M1 macrophage) and alternatively activated macrophage (M2 macrophage). It is well recognized that M1 macrophage is prominent in acute allograft rejection (32). To explore the effect of iTreg treatment on macrophage activity in the AMR model, we used fluorescence microscopy and flow cytometry to examine the phenotypes, frequencies, and numbers of macrophages in renal allografts obtained 5 days after transplantation. Macrophages were found to be the most prominent infiltrating immune cell type in grafts, and the M1 macrophage was the predominant macrophage type in this AMR model. Levels of total macrophages and specifically the M1 subset were found at significantly lower levels in iTreg-treated allografts relative to controlled grafts. Interestingly, there were no significant differences on the numbers of M2 macrophage between iTreg and control groups (Figures 5A,B). Flow cytometry analysis further validated the results of macrophage infiltration in the allografts observed from immunofluorescence (Figure 5C).

Given the fact that the M1 macrophage can affect allograft rejection through the release of pro-inflammatory cytokines such as iNOS and GM-CSF (32, 33), we also measured the levels of these pro-inflammatory cytokines in allografts. mRNA levels for iNOS and GM-CSF in the grafts were analyzed by qPCR. Compared with controls, iTreg treatment also significantly reduced the levels of iNOS and GM-CSF in allografts (Figure 5D), providing an additional evidence that iTreg have compromised M1 macrophage function in the AMR model.

Recently, greater attention has been paid to the roles of NK cells in AMR. Indeed, NK cells are clearly found in allografts at 5 days after transplantation in AMR model. Interestingly, we found that iTreg treatment significantly decreased the frequency and absolute numbers of NK cells in AMR relative to controls (Figures 5E–G).

### iTreg Treatment Decreases B Cell and Plasma Cell Infiltration in Grafts

**Figure 6:**
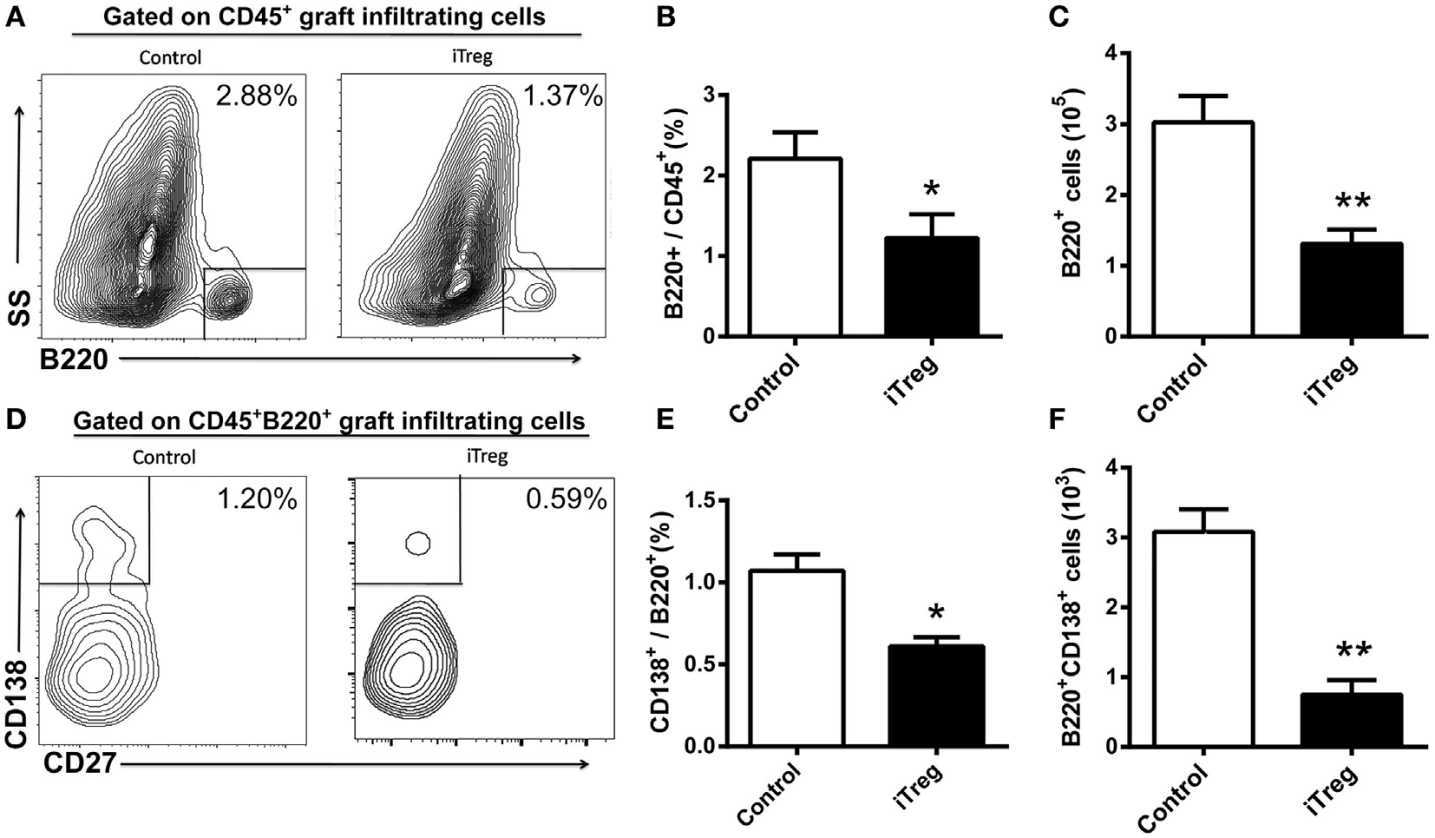
iTreg treatment reduces B cells and plasma cells infiltration in renal grafts. Renal allografts were obtained at 5 days post operation. **(A)** B220^+^ cells among CD45^+^ cells; **(B)** relative percentages of B220^+^ cells among the CD45^+^ cells; **(C)** absolute number of B220^+^ cells; **(D)** CD138^+^ cells among the total B220^+^ cells; **(E)** CD138^+^ cells among the B220^+^ cells, and **(F)** B220^+^CD138^+^ cells. Data are shown as mean ± SD of four transplants in each group (*p < 0.05, **p < 0.01).

Using flow cytometry, we determined that the frequency of infiltrating B cells (B220^+^) and plasma cells (B220^+^CD138^+^) was significantly less in the allografts obtained at 5 days after transplantation from iTreg treatment than upon treatment with PBS (Figures 6A,B,D,E). Accordingly, the absolute numbers of B220^+^ B cells and CD138^+^ plasma in the allografts of iTreg-treated mice were much lower than those in the control group (Figures 6C,F).

## Discussion

In this study, we confirmed for the first time that the distinctive ability of iTregs-induced *ex vivo* from CD4^+^CD25^−^ precursors to effectively attenuate organ injury and prolong renal allograft survival in a mouse model of acute AMR. iTreg treatment also resulted in a comprehensive immunoregulatory mechanism for preventing AMR, including the inhibition of DSA production and the reduction in the numbers as well as frequencies of multiple cell types found infiltrating allografts.

Fully MHC-mismatched kidney grafts were transplanted into presensitized recipients to establish a mouse model of acute AMR. We judged the disease activity in this model using serological and pathological evidence: serum DSA-IgG positivity, interstitial vasculitis, hemorrhage/edema, tubular necrosis and intragraft deposition of C3d. These parameters conform to Banff criteria (34). Many different studies have demonstrated that IgG is the major type of DSA which results in renal allograft rejection in animal models using skin presensitization methods. DSA-IgM levels are increased early after transplantation, probably as an indication of innate immunity (30, 35). In our model, the levels of IgM were not significantly higher in presensitized recipients, possibly through breakdown of serum IgM or due to its absorption to the renal graft at the time of examination. Furthermore, our comprehensive detection of cellular infiltrates in the grafts revealed that the major subsets of immune cells are macrophages and T cells, a finding that is in line with other studies (32, 36). However, we also observed NK cells, B cells, and plasma cells infiltrating the allografts, suggesting that these cells may also be involved in the pathogenesis of AMR.

The ability of nTreg expanded *ex vivo* to prevent rejection in skin allograft and transplant arteriosclerosis has been previously demonstrated (37, 38). iTregs share similar phenotypic and functional characteristics with nTreg. Because both the feasibility for the generation of sufficient numbers of iTregs and their inherent greater stability relative to nTreg, particularly under the condition of inflammation make them excellent choice for use in therapy, we choose *ex vivo* TGF-β-induced regulatory T cells to control immune responses in this study.

Our data demonstrated that iTreg therapy significantly alleviates renal allograft injury in AMR. Histological changes including peritubular capillaritis, tubular necrosis, C3d, and IgG deposition were clearly attenuated by iTreg treatment evaluated according to the Banff score criteria. TUNEL assay also showed less apoptotic cell deaths in allografts from mice with iTreg treatment. More importantly, iTreg treatment made at least 30% recipients to survive for a long time in the absence of any immunosuppression. This is surprising since multiple drugs used in combination are usually required to allow long-term survival of renal allografts in acute AMR (31).

This study, for the first time, demonstrates the ability of iTreg to decrease anti-MHC antibody production in the context of organ transplantation. We have previously demonstrated that treatment with iTreg significantly decreased IgG antibody levels and anti-dsDNA titers in lupus mice through the suppression of the B cell response (16). It was not known whether iTreg could decrease antidonor antibody in organ transplantation. To test this possibility, the level of DSA was examined at 5 days post transplantation. As expected, iTreg treatment decreased serum DSA-IgG levels and also the deposition of IgG in grafts. This could be the primary mechanism through which iTregs attenuate AMR since DSA is considered to be the major cause of AMR and is responsible for the destruction of renal allografts.

CD4^+^ T cells have been shown to play a key role in promoting renal allograft dysfunction in mixed AMR. CD4^+^ T cells not only facilitate antidonor antibody responses but also serve as T effectors that directly mediate graft injury without the involvement of host B cells, and depletion of CD4^+^ T cells prevents graft loss (39). Furthermore, memory CD4^+^ T cells were demonstrated to induce AMR of renal allografts (40). These studies revealed that CD4^+^ T cells are essential for AMR despite the observation that only a minor population of CD4^+^ T cells infiltrates the graft. In our model, the number of CD4^+^ T cells infiltrating in grafts was significantly reduced through iTreg treatment. Th1, Th17, and Tfh cell subsets were analyzed, and all were reduced through iTreg therapy. It is well recognized that Th1 cells and the cytokine interferon-γ play an important role in the pathogenesis of acute AMR (32, 41). High levels of Th17 cell infiltration were principally seen in the grafts by immunofluorescence staining, and IL-17 itself was found to be expressed in tubular epithelial cells in renal allograft with acute AMR, suggesting that Th17 may be involved in the pathogenesis of acute AMR and that IL-17 expression can result in tubular injury (42). The function of Tfh in renal allograft with AMR is less clear. Some studies suggest that Tfh cells and B cells can migrate to the allograft and are involved in DSA production, and that the presence of Tfh cells and B cells in renal allograft is associated with mixed rejection (43, 44). Foxp3^+^ cells were not increased in iTreg-treated grafts (Figure S2 in Supplementary Material), suggesting that injected iTreg did not migrate into renal allografts, it may work through the systemic immunoregulatory function.

Several studies have suggested that CD8^+^ T cells are not necessary in the pathogenesis of acute AMR since depletion of CD8^+^ T cells had no effect on renal allograft survival (30, 39). Nevertheless, we found the majority of T cells in the grafts at the time of rejection were indeed CD8^+^ T cells in this model, suggesting CD8^+^ T cells are indeed involved in the process of acute AMR. It is notable that iTreg treatment did not change total CD8^+^ cells but significantly reduced the frequency and number of Tc1 cells, CD8^+^IFNγ^+^ cells. Whether this resulted in a benefit to allograft survival will require further investigation.

Our previously published data and many other studies suggested that M1 macrophages and Th1 cytokines play an important role in the pathogenesis of AMR (32, 41, 45) and that macrophages are considered to be the most inflammatory cells in the grafts of this model. We now provided evidence showing the predominance of M1 over M2 macrophage infiltration in renal allografts in acute AMR, a finding that is consistent with our previous conclusions (41, 45). Our results also demonstrated that iTreg treatment markedly diminished absolute numbers of M1 macrophage but had no effect on M2 macrophage infiltration in the grafts with acute AMR. Moreover, iTreg also blocked the release of pro-inflammatory cytokines from M1 macrophages found in allograft in AMR. This may very well represent the primary mechanism through which graft rejection and injury are attenuated in this model.

The critical role of NK cells in acute AMR has been widely accepted. Hidalgo et al. used microarray-based gene profiling of biopsies from renal allografts with AMR and found the presence of transcripts associated with NK cells and their activation within the allograft (46). Moreover, NK1.1 mAb infusion, used to deplete NK cells, promoted long-term renal allograft survival in recipients with acute AMR (47). In this study, we observed moderate NK cell infiltration in grafts at the time of rejection and found that iTreg treatment markedly diminished the frequency and number of NK cells. Thus, we propose that iTreg cells can target NK cells to prevent allograft injury and rejection.

The functional repercussions of B cell and plasma cell infiltration in grafts are at present unclear. Several studies have suggested that they are involved in antidonor antibody production and that the presence of B cells and plasma cells resulted in the deterioration of allograft function (43, 48, 49). As discussed earlier, iTreg can both directly and indirectly suppress B cell and plasma cell activation. As expected, we found the percentages and numbers of B cells and plasma cells infiltrating in grafts were reduced through iTreg treatment. These data highlight the nature of B cell and plasma cell infiltration as critical parameters of AMR. Modulating B cell and plasma cell infiltration through iTreg treatment may contribute to the attenuation of allograft injury.

Overall, as a cell with comprehensive immunoregulatory abilities, we found that the iTreg modulated multiple immunological parameters that are involved in the process of AMR, eventually preventing allograft rejection. It cannot be ignored that iTreg treatment only prevents about 30% of allografts in current study. Further studies must consider altering cell dose, administration protocol, and/or combination with other immunosuppressive drugs. Despite the current limited success in preventing graft rejection, our findings provide new promise for the use of iTreg therapy in the prevention of AMR in patients undergoing kidney transplantation.

## Ethics Statement

This study was carried out in accordance with the recommendations of the animal use protocol, which was approved by the Institutional Animal Care and Use Committee of Sun Yat-Sen University (Approve Number: 160520).

## Author Contributions

QS and SZ designed the research; TL, YX, DZ, SL, ML, JC, DB, HZ, and YZ performed the experiments; DZ analyzed the data; TL, SZ, DB, and YX wrote the manuscript. All the authors read and approved the final manuscript.

## Conflict of Interest Statement

The authors declare that this research was conducted in the absence of any commercial or financial relationships that could be construed as a potential conflict of interest.
