# Supplementary figures and images for: In Vivo Attenuation of Antibody-Mediated Acute Renal Allograft Rejection by Ex Vivo TGF-β-Induced CD4+Foxp3+ Regulatory T Cells
Source: Front Immunol. 2017 Oct 16;8:1334. doi: 10.3389/fimmu.2017.01334 (PMC5650643; doi:10.3389/fimmu.2017.01334)

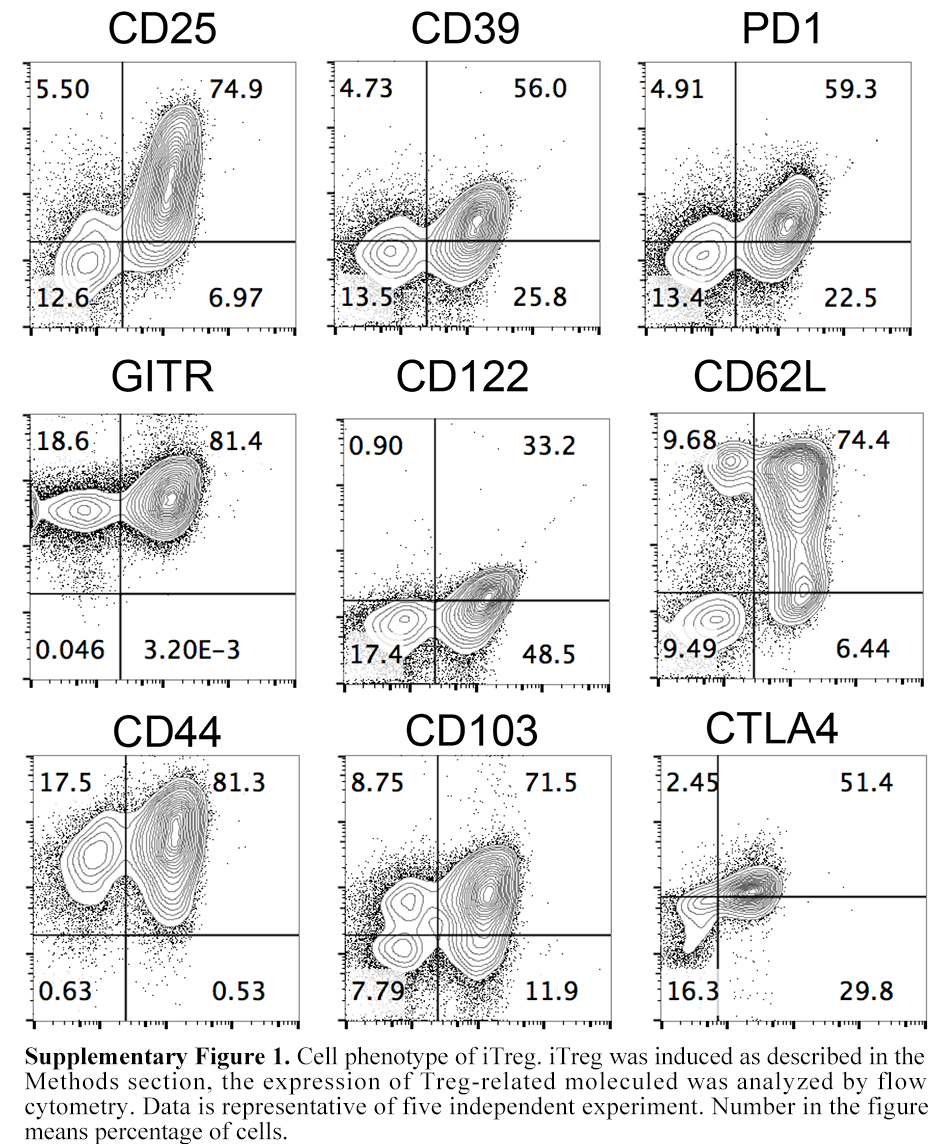

Supplement: Supplementary file 1 [file image_1.tif]

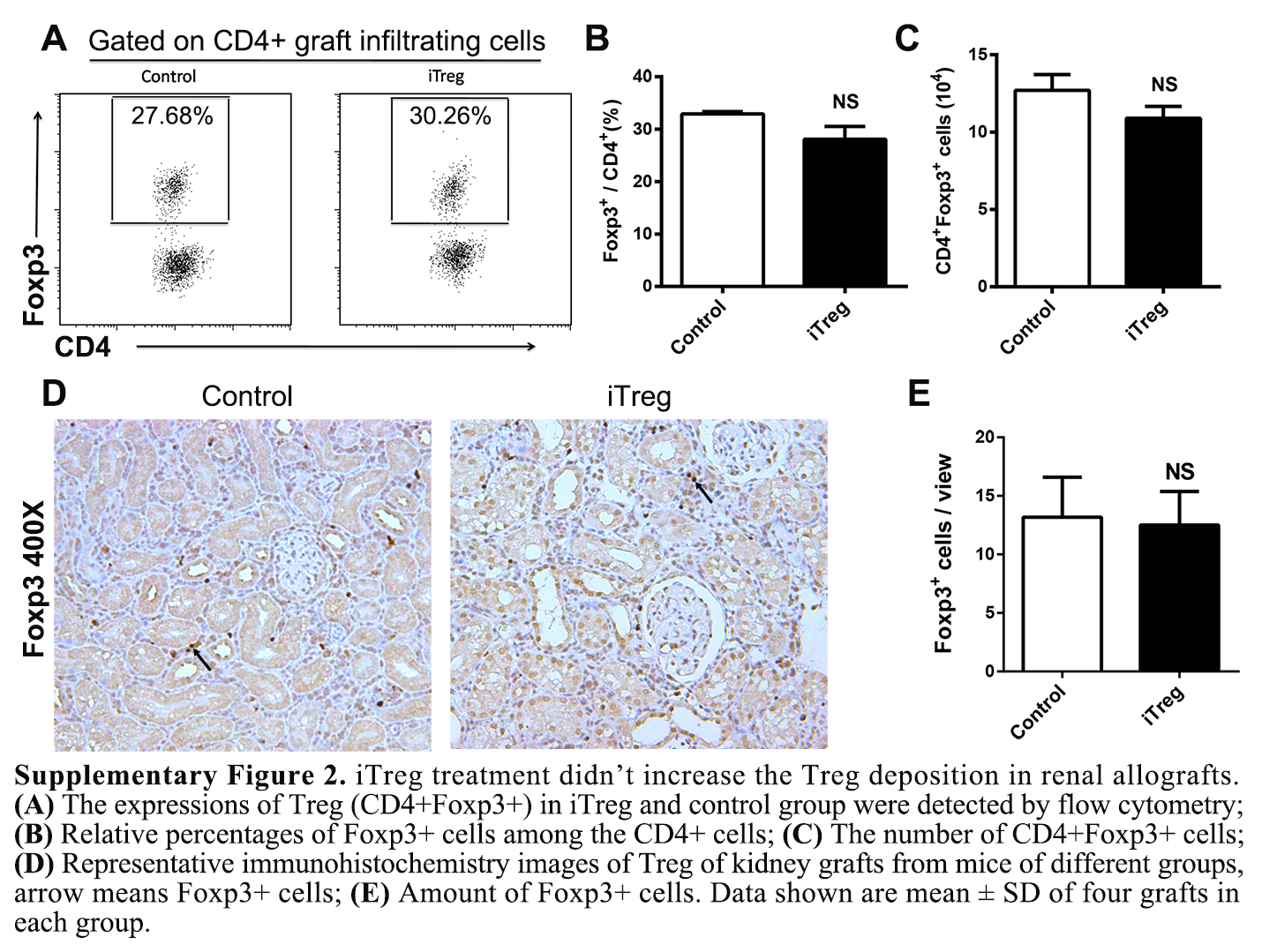

Supplement: Supplementary file 2 [file image_2.tif]
